# Supplementary material for: Elevated rates of autism, other neurodevelopmental and psychiatric diagnoses, and autistic traits in transgender and gender-diverse individuals
Source: Nat Commun. 2020 Aug 7;11:3959. doi: 10.1038/s41467-020-17794-1 (PMC7415151; doi:10.1038/s41467-020-17794-1)
Supplement: Supplementary file 1 — Supplementary Information [file 41467_2020_17794_MOESM1_ESM.pdf]

Elevated rates of autism, other neurodevelopmental and psychiatric diagnoses and autistic traits in transgender and gender-diverse individuals

Varun Warrier[1,5], David M. Greenberg[1,2], Elizabeth Weir[1], Clara Buckingham[1],

Paula Smith[1], Meng-Chuan Lai[1,3,4], Carrie Allison[1], and Simon Baron-Cohen[1,4]

1. Autism Research Centre, Department of Psychiatry, University of Cambridge; Douglas House, 18B Trumpington Road, Cambridge CB2 8AH
2. Interdisciplinary Department of Social Sciences and Department of Music, Bar-Ilan University, Ramat Gan, 5290002, Israel
3. Child and Youth Mental Health Collaborative, Centre for Addiction and Mental Health and The Hospital for Sick Children, Department of Psychiatry, University of Toronto; 80 Workman Way, Toronto, Ontario M6J 1H4, Canada
4. Department of Psychiatry, National Taiwan University Hospital and College of Medicine; No.7, Zhongshan South Rd., Taipei 10002, Taiwan
5. Correspondence: Varun Warrier (vw260@medschl.cam.ac.uk), or Simon Baron-Cohen (sb205@cam.ac.uk)

Supplementary Figure 1: Kernel density plot of scores on the four self-report measures in the C4 Dataset

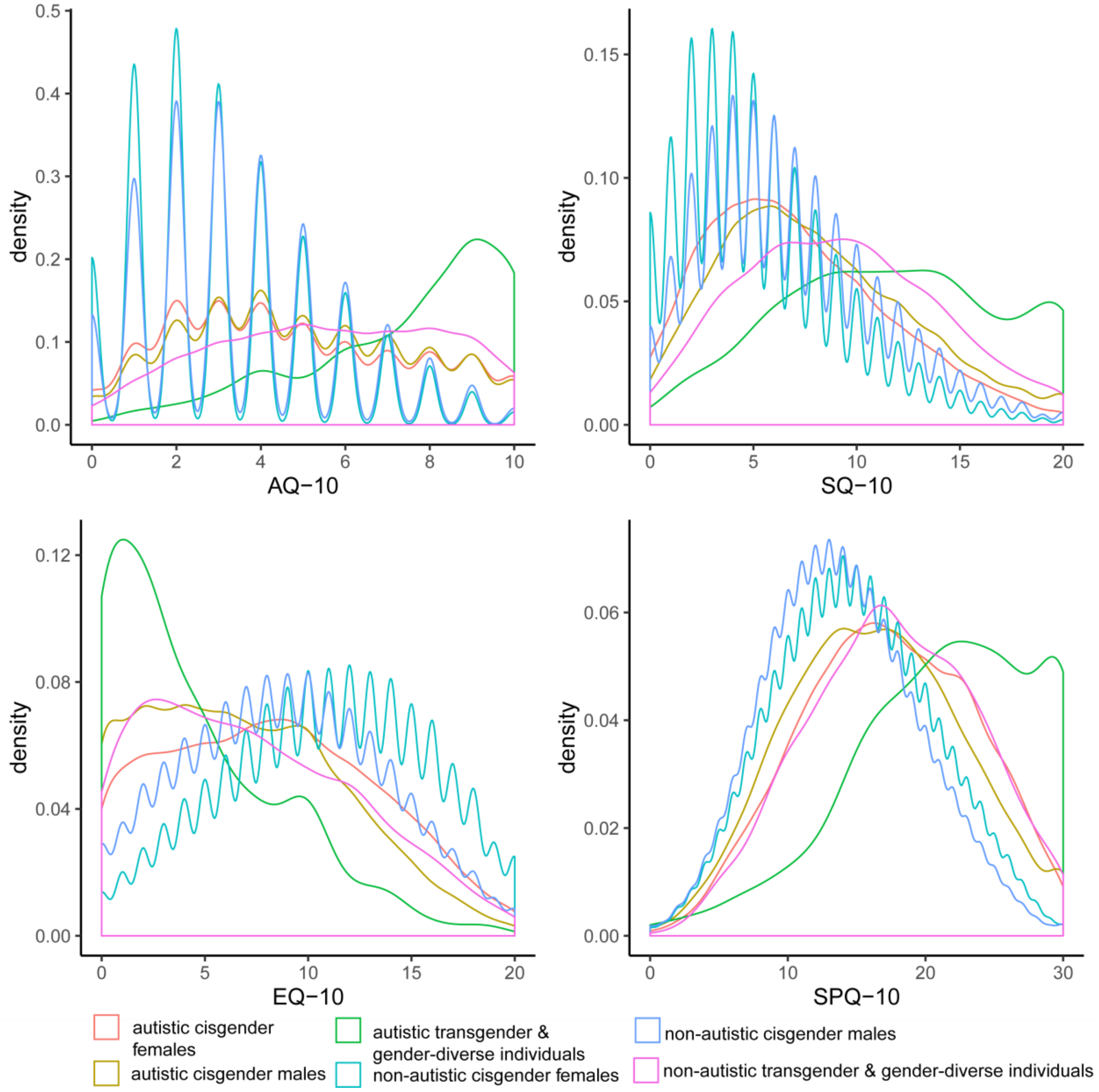

This figure provides kernel density plots for scores on the four self-report measures (AQ-10, EQ-10, SQ-10, and SPQ-10) for non-autistic participants from the C4 dataset based on their gender and autism diagnosis. Scales on the axes are different between the panels.

Supplementary Figure 2: Cumulative distribution Function of D-scores by gender and autism diagnosis in the C4 dataset

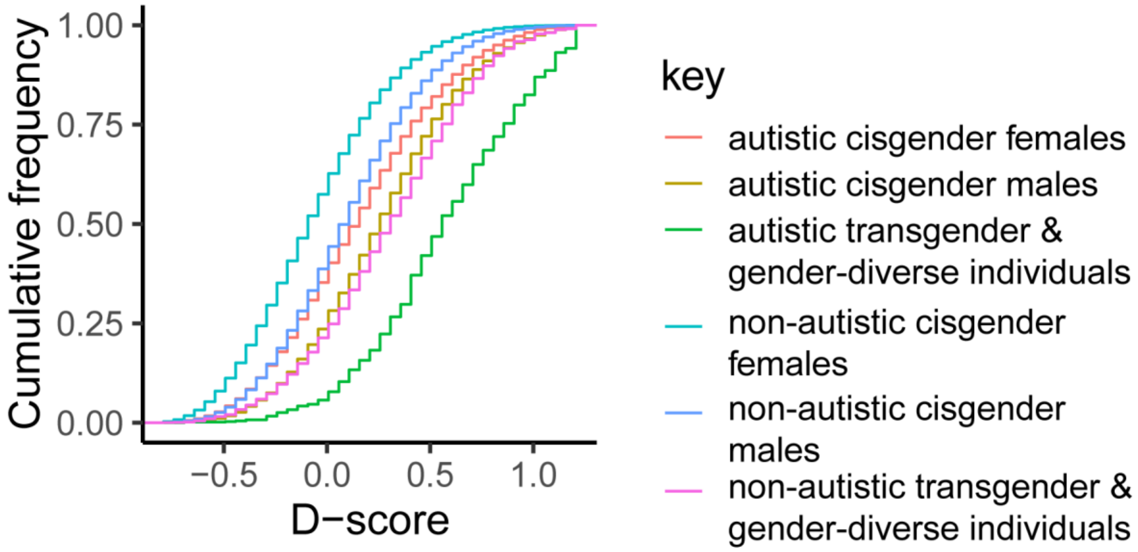

This figure provides the cumulative distribution function based on D-score (x-axis) by gender and autism diagnosis (coloured lines). D-scores were calculated only for the C4 dataset.

Supplementary Table 1: Participant demographics across all five datasets

	C4	MU	IMAGE	APHS	LifeLines
**Cisgender Males (autistic)**	193,398 (13,317)	42,957 (666)	994 (177)	766 (387)	15,275 (252)
**Cisgender Females (autistic)**	317,891 (13,934)	42,024 (365)	747 (153)	1,383 (562)	22,191 (184)
**Transgender and gender-diverse individuals (autistic)**	2,811 (668)	689 (55)	62 (36)	162 (133)	53 (3)
**Cisgender Males - Mean Age (SD)**	26.37 (10.86)	26.89 (12.70)	31.98 (13.67)	42.56 (15.81)	54.99 (12.71)
**Cisgender Females - Mean Age (SD)**	30.68 (12.34)	28.39 (12.67)	32.33 (16.12)	41.10 (14.88)	52.13 (12.63)
**Transgender and gender-diverse individuals - Mean Age (SD)**	25.44 (10.04)	22.38 (10.67)	29.67 (9.86)	35.18 (12.02)	47.88 (12.11)

This table provides the number of males, females, and transgender and gender-diverse individuals across the five datasets. Number of autistic individuals are provided in parenthesis. Mean age and standard deviation (in parenthesis) are also provided for all datasets by gender.

Supplementary Table 2: Educational attainment by gender identity for all four datasets

Category	Cisgender Males	Cisgender Females	Transgender and gender-diverse individuals
		**C4**	
**Did not complete High School (0)**	5,306	8,885	137
**High School (or A-levels) Diploma (1)**	10,936	17,942	361
**Undergraduate degree (2)**	77,800	118,583	1,154
**Postgraduate degree (3)**	77,679	121,103	849
**Prefer not to say (4)**	21,676	51,376	310
		**MU**	
**No high school**	1,083	751	22
**High school**	11,972	8,908	196
**Undergraduate**	13,450	14,251	175
**Still studying**	6,978	6,341	184
**Postgraduate**	9,357	11,536	107
**Missing**	117	137	5
		**IMAGE**	
**No high school**	12	18	3
**High school**	18	26	3
**Vocational**	22	19	4

**Undergraduate**	532	470	29
**Postgraduate**	405	205	21
**Missing**	5	9	2
**APHS**			
**No high school**	33	30	6
**High school**	131	235	23
**Vocational**	118	209	23
**Undergraduate**	210	414	55
**Postgraduate**	272	493	54
**Missing**	2	2	1
**LifeLines**			
**No education**	22	18	0
**Primary school**	113	152	1
**Lower or preparatory secondary**	1,492	1,443	3
**Junior general secondary**	1,426	2,709	5
**Secondary vocational**	4,573	6,555	10
**Senior general secondary**	1,167	2,495	7
**Higher vocational**	4,932	6,778	15
**University**	1,573	1,707	9

This table provides educational attainment for all three genders (in numbers) in all four datasets. Educational attainment was quantified differently across different datasets

Supplementary Table 3: OR and 95%CI for autism in transgender and gender-diverse individuals with males or females as the reference group.

Model	Dataset	Reference category	OR	UCI	LCI	p-value
**Model 1**	C4	Cisgender Males	4.21	4.6	3.85	<2E-16
		Cisgender Females	6.8	7.42	6.22	<2E-16
	MU	Cisgender Males	5.5	7.28	4.1	<2E-16
		Cisgender Females	9.92	13.2	7.32	<2E-16
	APHS	Cisgender Males	4.46	6.96	2.95	3.60E-13
		Cisgender Females	6.66	10.29	4.45	<2E-16
	IMAGE	Cisgender Males	6.39	10.93	3.75	6.32E-14
		Cisgender Females	5.35	9.24	3.14	5.23E-11
	LifeLines	Cisgender Males	3.63	11.73	1.12	0.02
		Cisgender Females	6.88	20.85	2.27	1.00E-04
**Model 2**	C4	Cisgender Males	3.88	4.25	3.54	< 2E-16
		Cisgender Females	5.31	5.82	4.85	< 2E-16
	MU	Cisgender Males	4.96	6.61	3.71	< 2E-16
		Cisgender Females	8.25	11.1	6.12	< 2E-16
	APHS	Cisgender Males	4.79	7.4	3.1	1.70E-12
		Cisgender Females	6.86	10.49	4.49	< 2E-16
	IMAGE	Cisgender Males	4.64	9.01	2.39	5.41E-06
		Cisgender Females	12.04	24.56	5.9	7.51E-12
	LifeLines	Cisgender Males	1.86	0.44	7.8	0.44
		Cisgender Females	4.69	19.83	1.1	0.035

This table provides the Odds Ratios (OR), and upper (UCI) and lower (LCI) 95% Confidence Intervals for autism in transgender and gender-diverse individuals in the five datasets. The reference or baseline category was either males or females. Model 1 was a χ^2 test and Model 2 was a regression accounting for age and educational attainment. p-values for the ORs are also provided.

Supplementary Table 4: Sensitivity Analyses in the MU cohort after splitting the data into four gender indicators (Males, Females, Transgender and Others)

Comparison	OR	95%CI	p-value
**Females vs Males**	0.60	0.52 - 0.68	1.34E-14
**Transgender vs Males**	6.22	4.34 - 8.91	< 2E-16
**Transgender vs Females**	10.35	7.17 - 14.94	< 2E-16
**Others vs Males**	3.65	2.30 - 5.79	3.79E-08
**Others vs Females**	6.07	3.80 - 9.69	3.84E-14
**Transgender vs Others**	0.58	1.04 - 0.33	0.068

This table provides the results of the sensitivity analyses in the MU dataset. Odds Ratios (ORs) for autism were calculated using linear regression after accounting for age and educational attainment. 95% Confidence Intervals (95%CI) and p-values for the ORs are also provided. We conducted 6 different comparisons, with the reference category provided later. For example, in Females vs Males, we calculated the ORs for females using males as the reference category. All the p-values were significant except for the Transgender vs Other comparison.

Supplementary Table 5: Mean scores and standard deviations for the four measures by gender and autism diagnosis in the C4 dataset

	Non-autistic individuals			Autistic individuals		
**Questionnaire**	Cisgender Males	Cisgender Females	Transgender and gender-diverse individuals	Cisgender Males	Cisgender Females	Transgender and gender-diverse individuals
**AQ-10**	3.56 (2.28)	3.17 (2.23)	5.56 (2.69)	4.88 (2.67)	4.72 (2.77)	7.44 (2.38)
**EQ-10**	8.88 (4.74)	10.79 (4.85)	7.32 (5.03)	6.91 (4.71)	8.21 (5.05)	4.44 (4.19)
**SQ-10**	6.71 (4.17)	5.44 (3.88)	9.19 (4.73)	8.05 (4.63)	7.18 (4.42)	11.75 (5.27)
**SPQ-10**	13.98 (5.48)	14.80 (5.74)	17.52 (6.12)	16.28 (6.27)	17.18 (6.21)	21.37 (6.69)

This table provides the mean scores and standard deviations (in parenthesis) for cisgender males, cisgender females, and transgender and gender-diverse individuals for four measures in the C4 dataset. Means and standard deviations were calculated separately for autistic and non-autistic individuals.

Supplementary Table 6: Cohen's D for gender comparisons for the four measures across autistic and non-autistic individuals

	Non-autistic individuals			Autistic individuals			
**Questionnaire**	Males vs Females	Transgender and gender-diverse vs Males	Transgender and gender-diverse vs Females	Males vs Females	Transgender and gender-diverse vs Males	Transgender and gender-diverse vs Females	Transgender and gender-diverse: autistic vs non-autistic
**AQ-10**	0.17	0.80	0.96	0.05	1.01	1.05	0.74
**EQ-10**	0.39	0.32	0.70	0.26	0.55	0.81	0.62
**SQ-10**	0.31	0.55	0.86	0.19	0.74	0.93	0.54
**SPQ-10**	0.14	0.61	0.46	0.14	0.78	0.64	0.60

This table provides the Cohen's D for all gender-based comparisons in the C4 data for all four questionnaires. We conducted gender-based comparisons (t-tests) separately in non-autistic and in autistic individuals. Additionally, we also conducted autism vs non-autism comparisons only for transgender and gender-diverse individuals for all four measures in the C4 dataset. All comparisons are statistically significant at p-value < 2.2E-16.

Supplementary Table 7: Brain-types for autistic and non-autistic individuals in the C4 dataset

	Non-autistic Cisgender Males	Non-autistic Cisgender Females	Non-autistic Transgender and gender-diverse individuals	Non-autistic Cisgender Males	Non-autistic Cisgender Females	Non-autistic Transgender and gender-diverse individuals
**Extreme S**	4.00	1.68	13.15	11.06	7.94	34.73
**S**	40.29	25.56	53.01	51.43	42.37	51.79
**B**	31.20	29.80	20.34	24.03	26.95	9.73
**E**	23.80	40.03	13.06	13.20	21.81	3.59
**Extreme E**	0.69	2.91	0.41	0.26	0.91	0.15

This table provides the percentage of individuals by their brain types for all three genders groups separately in autistic and non-autistic individuals in the C4 dataset.

Supplementary Table 8: ORs for six different neurodevelopmental and psychiatric conditions and autism in transgender and gender-diverse individuals compared to cisgender individuals in two datasets

	Model 1				Model 2				Model 3			
						C4						
	**OR**	**UCI**	**LCI**	**p-value**	**OR**	**UCI**	**LCI**	**p-value**	**OR**	**UCI**	**LCI**	**p-value**
**Autism**	5.53	6.04	5.06	<2E-16	4.59	5.03	4.20	<2E-16	NA	NA	NA	NA
**ADHD**	6.56	7.21	5.98	<2E-16	5.76	6.34	5.24	<2E-16	4.54	5.15	4.01	<2E-16
**Depression**	3.86	4.16	3.59	<2E-16	4.01	4.32	3.72	<2E-16	4.15	4.52	3.81	<2E-16
**Bipolar**	5.23	5.99	4.57	<2E-16	4.83	5.54	4.22	<2E-16	4.98	5.83	4.26	<2E-16
**Learning Disorder**	3.48	3.91	3.09	<2E-16	3.08	3.47	2.74	<2E-16	1.92	2.31	1.64	1.13E-14
**OCD**	5.18	5.82	4.61	<2E-16	4.69	5.27	4.17	<2E-16	2.84	3.36	2.39	<2E-16
**Schizophrenia**	28.52	33.66	24.17	<2E-16	19.73	23.42	16.62	<2E-16	6.39	9.50	4.30	<2E-16
	**Model 1**				**Model 2**				**Model 3**			
						**MU**						
	**OR**	**UCI**	**LCI**	**p-value**	**OR**	**UCI**	**LCI**	**p-value**	**OR**	**UCI**	**LCI**	**p-value**

**Autism**	6.82	5.10	8.94	<2E-16	5.82	4.39	7.73	<2E-16	NA	NA	NA	NA
**ADHD**	2.25	2.94	1.72	3.33E-09	2.07	2.71	1.58	1.20E-07	1.91	2.58	1.41	2.49E-05
**Depression**	3.84	4.57	3.22	<2E-16	3.89	4.64	3.26	<2E-16	3.91	4.72	3.25	< 2E-16
**Bipolar**	2.59	3.67	1.83	7.67E-08	2.56	3.64	1.81	1.28E-07	2.38	3.51	1.62	1.18E-05
**OCD**	2.54	3.65	1.77	4.55E-07	2.35	3.38	1.63	4.16E-06	2.18	3.29	1.45	1.80E-04
**Schizophrenia**	2.15	5.81	0.80	1.31E-01	1.81	4.91	0.67	2.46E-01	1.11	4.48	0.27	8.88E-01

This table provides the Odds Ratios and 95 % upper (UCI) and lower (LCI) confidence intervals and the p-value for six different neurodevelopmental and psychiatric conditions in transgender and gender-diverse individuals compared to cisgender individuals. Three models were conducted. Model 1 is a χ^2 test. Model 2 is a logistic regression after accounting for age and educational attainment. Model 3 is Model 2 repeated in the subset of participants without an autism diagnosis. For comparison, we provide the ORs and 95% CIs for autism for Models 1 and 2. Model 3 cannot be conducted for autism. The table provides the results from both the C4 and the MU datasets.

Supplementary Table 9: ORs for six different neurodevelopmental and psychiatric conditions and autism in transgender and gender-diverse individuals compared to males and females separately in two datasets

C4

	Reference	OR	UCI	LCI	p-value	OR	UCI	LCI	p-value	OR	UCI	LCI	p-value
**Autism**	Cisgender Male	4.21	4.60	3.85	<2E-16	3.88	4.25	3.54	<2E-16	NA	NA	NA	NA
	Cisgender Female	6.80	7.42	6.22	<2E-16	5.31	5.82	4.85	<2E-16	NA	NA	NA	NA
**ADHD**	Cisgender Male	4.77	5.24	4.34	<2E-16	4.39	4.83	3.99	<2E-16	3.50	3.97	3.09	<2E-16
	Cisgender Female	8.47	9.31	7.70	<2E-16	7.38	8.13	6.71	<2E-16	5.67	6.43	5.01	<2E-16
**Depression**	Cisgender Male	6.41	6.91	5.94	<2E-16	6.31	6.81	5.85	<2E-16	6.64	7.24	6.09	<2E-16
	Cisgender Female	3.01	3.25	2.80	<2E-16	3.11	3.35	2.88	<2E-16	3.23	3.52	2.96	<2E-16
**Bipolar**	Cisgender Male	7.50	8.62	6.53	<2E-16	6.87	7.90	5.97	<2E-16	7.11	8.36	6.06	<2E-16
	Cisgender Female	4.42	5.06	3.86	<2E-16	4.00	4.58	3.48	<2E-16	4.14	4.85	3.54	<2E-16
**Learning Disorder**	Cisgender Male	3.28	3.70	2.92	<2E-16	3.09	3.48	2.74	<2E-16	2.01	2.38	1.69	1.32E-15
	Cisgender Female	3.62	4.07	3.22	<2E-16	3.09	3.48	2.74	<2E-16	1.92	2.27	1.62	7.49E-14
**OCD**	Cisgender Male	7.47	8.42	6.63	<2E-16	6.86	7.74	6.08	<2E-16	4.58	5.45	3.85	<2E-16
	Cisgender Female	4.36	4.90	3.88	<2E-16	3.82	4.29	3.39	<2E-16	2.25	2.66	1.89	<2E-16
**Schizophrenia**	Cisgender Male	18.08	21.48	15.22	<2E-16	13.80	16.48	11.56	<2E-16	5.22	7.81	3.49	<2E-16
	Cisgender Female	43.94	52.60	36.71	<2E-16	29.56	35.62	24.53	<2E-16	7.68	11.49	5.13	<2E-16

				MU									
	**Reference**	**OR**	**UCI**	**LCI**	**p-value**	**OR**	**UCI**	**LCI**	**p-value**	**OR**	**UCI**	**LCI**	**p-value**
**Autism**	Cisgender Male	5.3	6.99	3.96	<2E-16	4.75	3.57	6.32	<2E-16	NA	NA	NA	NA
	Cisgender Female	9.6	12.74	7.1	<2E-16	7.94	5.92	10.66	<2E-16	NA	NA	NA	NA
**ADHD**	Cisgender Male	1.99	2.6	1.52	6.30E-07	1.87	2.45	1.42	6.20E-06	1.72	2.21	1.34	4.30E-04
	Cisgender Female	2.61	3.43	1.99	4.20E-12	2.37	3.12	1.8	5.90E-10	2.18	2.96	1.61	4.80E-07
**Depression**	Cisgender Male	5.18	6.18	4.34	<2E-16	5.31	6.35	4.45	<2E-16	5.46	6.59	4.52	<2E-16
	Cisgender Female	2.91	3.47	2.44	<2E-16	2.88	3.44	2.41	<2E-16	2.87	3.46	2.38	<2E-16
**Bipolar**	Cisgender Male	3.01	4.28	2.12	8.40E-10	3.04	4.33	2.13	7.50E-10	2.87	4.25	1.94	1.40E-07
	Cisgender Female	2.25	3.2	1.59	5.60E-06	2.16	3.07	1.51	1.90E-05	1.98	2.93	1.34	6.20E-04
**OCD**	Cisgender Male	3.3	4.76	2.28	2.00E-10	3.1	4.48	2.14	2.00E-09	3.02	4.57	1.99	1.70E-07
	Cisgender Female	2.03	2.92	1.41	1.40E-04	1.82	2.62	1.26	1.30E-03	1.64	2.48	1.09	1.70E-02
**Schizophrenia**	Cisgender Male	1.86	5.05	0.68	0.22	1.68	4.58	0.61	3.10E-01	1	4.09	0.25	9.90E-01
	Cisgender Female	2.59	7.11	0.95	0.06	2.02	5.56	0.73	1.70E-01	1.27	5.23	0.31	7.30E-01

This table provides the Odds Ratios and 95 % upper (UCI) and lower (LCI) confidence intervals and the p-value for six different neurodevelopmental and psychiatric conditions in transgender and gender-diverse individuals compared to males and females (reference category). Three models were conducted. Model 1 is a χ^2 test. Model 2 is a logistic regression after accounting for age

and educational attainment. Model 3 is Model 2 repeated in the subset of participants without an autism diagnosis. For comparison, we provide the ORs and 95% CIs for autism for Models 1 and 2. Model 3 cannot be conducted for autism. The table provides the results from both the C4 and the MU datasets.

Supplementary Table 10: Results of multiple regression analyses for selected neurodevelopmental and psychiatric conditions

	C4			MU		
	Beta	SE	p-value	Beta	SE	p-value
**Autism**	1.23	0.05	2.00E-16	1.37	0.18	2.77E-14
**ADHD**	1.13	0.06	2.00E-16	0.26	0.15	0.09
**Bipolar**	1.14	0.07	2.00E-16	0.35	0.19	0.07
**Depression**	1.27	0.04	2.00E-16	1.25	0.10	2.00E-16
**LD**	0.42	0.07	1.60E-08	NA	NA	NA
**OCD**	0.47	0.07	3.90E-10	0.19	0.21	0.37
**Schizophrenia**	0.67	0.12	3.20E-08	-0.11	0.52	0.83

This table provides the results of the multiple regression analyses in the C4 and MU datasets. The reference gender group is cisgender individuals, and the tested gender group is transgender and gender-diverse individuals.

Supplementary Table 11: Details of tests conducted

**Aim 1**	**N**
Transgender and gender-diverse vs Males/Females/cisgender in 5 datasets (Chi square)	15
Transgender and gender-diverse vs Males/Females/cisgender in 5 datasets (regression)	15
MU sensitivity analyses	6
**Aim 2**	
Transgender and gender-diverse vs Males/Females for 4 self-report measures (T test) in autistic and non-autistic individuals	16
Transgender and gender-diverse vs Males/Females for 4 self-report measures (Linear regression)	8
Transgender and gender-diverse vs Males/Females (IMAGE and LifeLines)	4
Autistic transgender and gender-diverse vs non-autistic transgender and gender-diverse (C4)	4
Brain Type	18
**Aim 3**	
Transgender and gender-diverse vs Males/Females/Cis (3 models and 5 conditions)	54
Transgender and gender-diverse vs Males/Females/Cis (3 models and 4 conditions)	36
Exploratory analysis: Suspected autism – Transgender and gender-diverse vs Males/Females	3
**Total**	**179**

This table provides the list of all statistical analyses conducted.

Supplementary Methods: Power calculations

We conducted power calculations in the LifeLines cohort given the low number of transgender and gender-diverse individuals. Effect sizes were obtained from the C4 dataset. We conducted power calculations separately for males, females, and cisgender gender groups.

X^2 tests

Parameters	Males	Females	Cisgender
Proportion p2	0.003273	0.002253	0.00134
Proportion p1	0.011905	0.016304	0.006881
alpha error	0.05	0.05	0.05
Sample size group 1	255	187	439
Sample size group 2	15325	22241	37536
Power achieved	0.54	0.78	0.7
N for 80% power	34799	20436	56661

Logistic regression

Parameters	Males	Females	Cisgender
Tails	2	2	2
Odds ratio	4.21	6.8	5.33
Pr(Y = 1|X = 1)H0	0.0032	0.0022	0.00133
alpha error probability	0.05	0.05	0.05
Power	80	80	80
Distribution	Binomial	Binomial	Binomial
X param pi	0.016	0.008	0.011
Achieved power	0.62	0.69	0.66
N for 80% power	29,815	36524	66113

Linear regression

Parameter	Males	Females	Cisgender
Effect size(f2 based on incremental r2)	0.011	0.009	0.005
N	15580	22428	37975
Power	1	1	1
