# Supplementary material for: Elevated rates of autism, other neurodevelopmental and psychiatric diagnoses, and autistic traits in transgender and gender-diverse individuals
Source: Nat Commun. 2020 Aug 7;11:3959. doi: 10.1038/s41467-020-17794-1 (PMC7415151; doi:10.1038/s41467-020-17794-1)
Supplement: Supplementary file 3 — Reporting Summary [file 41467_2020_17794_MOESM3_ESM.pdf]

nature research

Corresponding author(s): Warrier

Last updated by author(s): May 28, 2020

Reporting Summary

Nature Research wishes to improve the reproducibility of the work that we publish. This form provides structure for consistency and transparency in reporting. For further information on Nature Research policies, see Authors & Referees and the Editorial Policy Checklist.

Statistics

For all statistical analyses, confirm that the following items are present in the figure legend, table legend, main text, or Methods section.

n/a	Confirmed	
☐	☒	The exact sample size (n) for each experimental group/condition, given as a discrete number and unit of measurement
☐	☒	A statement on whether measurements were taken from distinct samples or whether the same sample was measured repeatedly
☐	☒	The statistical test(s) used AND whether they are one- or two-sided<br>*Only common tests should be described solely by name; describe more complex techniques in the Methods section.*
☐	☒	A description of all covariates tested
☐	☒	A description of any assumptions or corrections, such as tests of normality and adjustment for multiple comparisons
☐	☒	A full description of the statistical parameters including central tendency (e.g. means) or other basic estimates (e.g. regression coefficient) AND variation (e.g. standard deviation) or associated estimates of uncertainty (e.g. confidence intervals)
☐	☒	For null hypothesis testing, the test statistic (e.g. F, t, r) with confidence intervals, effect sizes, degrees of freedom and P value noted<br>*Give P values as exact values whenever suitable.*
☒	☐	For Bayesian analysis, information on the choice of priors and Markov chain Monte Carlo settings
☐	☒	For hierarchical and complex designs, identification of the appropriate level for tests and full reporting of outcomes
☐	☒	Estimates of effect sizes (e.g. Cohen's d, Pearson's r), indicating how they were calculated

Our web collection on statistics for biologists contains articles on many of the points above.

Software and code

Policy information about availability of computer code

Data collection	No software was used for data collection.
Data analysis	Scripts are provided at: https://github.com/autism-research-centre/Atypical-gender-and-autism. All analyses were conducted in R version 3.4.4 (2018-03-15)

For manuscripts utilizing custom algorithms or software that are central to the research but not yet described in published literature, software must be made available to editors/reviewers. We strongly encourage code deposition in a community repository (e.g. GitHub). See the Nature Research guidelines for submitting code & software for further information.

Data

Policy information about availability of data

All manuscripts must include a data availability statement. This statement should provide the following information, where applicable:

- Accession codes, unique identifiers, or web links for publicly available datasets
- A list of figures that have associated raw data
- A description of any restrictions on data availability

As participants did not consent for their data to be publicly shared, even anonymized, data will be made available to only potential collaborators with ethical approval after they submit a research proposal to the Autism Research Centre, University of Cambridge, UK. Data for the LifeLines can be accessed at https://www.lifelines.nl/researcher. Research enquiries to access the Musical Universe Data can be made at https://musicaluniverse.io/contact/. For all other datasets, please contact the corresponding authors.

Field-specific reporting

Please select the one below that is the best fit for your research. If you are not sure, read the appropriate sections before making your selection.

☐ Life sciences ☒ Behavioural & social sciences ☐ Ecological, evolutionary & environmental sciences

For a reference copy of the document with all sections, see nature.com/documents/nr-reporting-summary-flat.pdf

Behavioural & social sciences study design

All studies must disclose on these points even when the disclosure is negative.

Study description	Quantitative. It is unclear whether transgender and gender-diverse individuals have elevated rates of autism diagnosis or traits related to autism compared to cisgender individuals in large non-clinic-based datasets. To investigate this, we use five independently recruited cross-sectional datasets consisting of 641,860 individuals who completed information on gender, neurodevelopmental and psychiatric diagnoses including autism, and measures of traits related to autism (self-report measures of autistic traits, empathy, systemizing, and sensory sensitivity).
Research sample	We used five datasets for this study. The largest of these (Channel 4 dataset) consists of N = 514,100 individuals who completed online questionnaires as a part of a Channel 4 television program about autism. These participants self-reported their autism diagnosis, and indicated their gender based on three options 'Male', 'Female' and 'Transgender'. To address autism-related self-selection bias in this dataset, we used a second dataset (Musical Universe, N = 85,670) recruited through a website for research about musical behavior, personality and cognition. Participants completed information about their autism diagnosis and selected their gender from four options: 'Male', 'Female', 'Transgender' and 'Other'. However, neither of these two datasets have separately recorded information on sex at birth and gender, and in both datasets, participants were asked to choose their 'Sex', although we acknowledge that the information collected is primarily of gender. To address this, we used two additional datasets where information was collected separately for sex at birth and gender. In the third dataset (APHS, N = 2,312), participants were recruited for an internet-based physical health survey. Participants completed information on their autism diagnosis including when they were diagnosed and who diagnosed them, their sex at birth, and their current gender identity. The fourth dataset (IMAGE, N = 1,803) consists of participants who were recruited for a genetic study of autism and mathematical ability. Participants completed information on their autism diagnosis, their sex at birth, and their gender. In addition, all autistic participants provided a copy of their diagnostic report to verify their diagnosis. The fifth and final dataset consists of a subset of participants from the LifeLines Cohort and Biobank73 (N = 37,975) who provided information on sex assigned at birth and gender, autism diagnosis, and completed a measure of autistic traits. This dataset consists of individuals who are considerably older than those in the other four datasets, and who were recruited primarily through GP clinics. None of the five datasets were recruited specifically to investigate the association between gender diversity and autism, which limits gender-based self-selection bias. As participants did not consent for their data to be publicly shared, even anonymized, data will be made available to only potential collaborators with ethical approval after they submit a research proposal to the Autism Research Centre, University of Cambridge, UK for four of the datasets (C4, MU, IMAGE, and APHS). Data for LifeLines can be obtained by making an application to the LifeLines Biobank (https://www.lifelines.nl/researcher). Research enquiries to access the Musical Universe Data can be made at https://musicaluniverse.io/contact/. A reporting summary for this Article is available as a Supplementary Information file.
Sampling strategy	Cross-sectional convenience sampling. As this was a convenience sampling, a priori power calculations were not conducted for all but the LifeLines data for the analyses. However, these datasets are large, and we used 5 different datasets to investigate the research questions and validate the results.
Data collection	Online questionnaire completion. Participants completed all questionnaires at home on their computer, or other devices. Blinding was not done as cases and controls were defined based on a priori hypotheses and information provided by the participants.
Timing	Ongoing data collection as it's based on convenience sampling. Data collected upto November 2019 used in the analyses. Data has been collected since 2006.
Data exclusions	Each dataset has specific data exclusion criteria which is indicated in the manuscript. Channel 4 dataset: overview<br>The Channel 4 dataset (C4 dataset) comprises participants who completed self-report measures as a part of the Channel 4 documentary titled "Are you autistic?", in Spring 2017A total of 758,916 entries were recorded. Participants provided information on demographics (gender (see below for details), age, educational attainment, geographical region, handedness, occupation, autism and other neurodevelopmental or psychiatric diagnosis) and completed four self-report measures. Participants who consented to share their data for research were asked: "Have you taken this survey before? To make sure our data are as accurate and as useful as possible please tell us if you've taken this survey before." If participants indicated that they had taken the survey before, they were marked as duplicates. After removing duplicates, we were left with a total of 695,166 participants. We were unable to use IP addresses to identify duplicates due to ethical constraints. We included participants aged 15 to 90 years, in line with previous research. Participants were asked to indicate their "Sex" using one of four options: "Male", "Female", "Transgender" and "Prefer not to say". Whilst "Sex" was asked in the survey, we recognize that the information provided here is of sex or gender, or both and we refer to this as gender throughout the manuscript. Whilst designing the survey we did not make a distinction between gender and sex as these terms are often used interchangeably in the general population. We further removed individuals who did not provide information on gender ("Prefer not to say"), resulting in N = 675,360 individuals.<br><br>LifeLines: Overview of dataset<br>The LifeLines Cohort is a Netherlands-based population cohort study, recruited between 2006 and 201379. Participants were invited through their general practitioners in three northern provinces in the Netherlands (Freisland, Groningen, and Drenthe). Notably,

participants were not invited if they had a severe mental health condition, which suggests that this dataset will be biased towards healthy participants. A total of 167,729 participants aged between 6 months to 93 years completed the baseline survey. The LifeLines dataset used in this study consists of 37,975 individuals from the cohort, who responded to an online questionnaire on autistic traits in summer 2019. All participants were at least 18 years of age. The participants in the LifeLines cohort were, on average, about twice as old as the participants in the C4 and the MU cohorts, and this may in part explain the relatively low number of transgender and gender-diverse individuals in this dataset. In addition, 37,574 participants provided information on their highest level of educational attainment (Supplementary Table 2).

The IMAGE study: Overview of dataset
The Investigating Mathematics and Autism using Genetics and Epigenetics (IMAGE) dataset consists of individuals recruited into a genetic study of autism and mathematical ability. This was done using two different research designs. The first targeted autistic and non-autistic individuals as a part of a case-control design (Nfinal = 292) by advertising in research databases, autism-related magazines, and on social media. The second targeted individuals who studied or were studying mathematics or a related degree (Nfinal = 1,803) by advertising in universities, mathematics societies, in mathematics specific or alumni magazines, or on social media. Participants registered at a bespoke website and provided contact details, demographics, and completed various questionnaires. As participants provided both their names and their contact details, we used this information to remove duplicate records.

Autism Physical Health Survey: Overview of dataset
The Autism Physical Health Survey (APHS) dataset consists of 2,312 individuals aged 16 – 90 years who were recruited via the Cambridge Autism Research Database (CARD), autism charities and support groups, and social media as a part of a study investigating the association between autism and physical health conditions. The study employed an anonymous, online self-report survey via Qualtrics. Participants were asked questions regarding their demographics, lifestyle factors (including diet, exercise, sleep, and sexual/social history), personal medical history, and family medical history for all first-degree, biological relatives. As the study was anonymous (as we did not collect IP addresses), we excluded records that we determined were likely to be duplicates. We excluded all records that matched a previous record across 11 categories: whether or not they had an autism diagnosis, specific autism diagnosis, type of practitioner who diagnosed them, year of diagnosis, syndromic autism (if applicable), country of residence, sex assigned at birth, current gender identity, age, maternal age at birth, paternal age at birth, and educational attainment.

Musical Universe dataset: Overview of dataset
The Musical Universe (MU) dataset consists of a total of 89,218 individuals who completed measures on musical behavior, personality, and cognition, in exchange for feedback about their scores at www.musicaluniverse.org. We identified duplicates first using IP addresses, and then, among individuals with identical IP addresses, using demographic variables – gender (see below for further information about this), age, educational attainment, occupation, and diagnosis. A total of 85,670 unique records were identified. Participants ranged in age from 18 to 88 years old (Supplementary Table 1).

Non-participation

Convenience sampling, so non-participation is not applicable.

Randomization

No randomization. In all five datasets, we investigated if rates of autism diagnosis significantly differed by gender by first conducting χ^2 tests (Model 1, unadjusted), and then by conducting logistic regressions adjusted for age and educational attainment as covariates (Model 2, adjusted). Both age and educational attainment were associated with autism diagnosis, with younger individuals more likely to receive an autism diagnosis80,81, and educational attainment typically negatively correlated with autism51. Further, these two variables were measured across all five datasets. In addition, for the IMAGE dataset, we included a dummy variable for the two studies participants were drawn from (mathematical ability and case-control) to account for potential confounding effects of recruitment.

Reporting for specific materials, systems and methods

We require information from authors about some types of materials, experimental systems and methods used in many studies. Here, indicate whether each material, system or method listed is relevant to your study. If you are not sure if a list item applies to your research, read the appropriate section before selecting a response.

Materials & experimental systems

n/a	Involved in the study	
☒	☐	Antibodies
☒	☐	Eukaryotic cell lines
☒	☐	Palaeontology
☒	☐	Animals and other organisms
☐	☒	Human research participants
☒	☐	Clinical data

Methods

n/a	Involved in the study	
☒	☐	ChIP-seq
☒	☐	Flow cytometry
☒	☐	MRI-based neuroimaging

Human research participants

Policy information about studies involving human research participants

Population characteristics

All are adults from five different datasets who completed a number of online questionnaires. Detailed population characteristics provided in the manuscript.

Recruitment	Each dataset had specific recruitment criteria. A convenience sampling framework was used in this study.
Ethics oversight	University of Cambridge Psychology Research Ethics Committee

Note that full information on the approval of the study protocol must also be provided in the manuscript.
